# A G-Protein β Subunit, AGB1, Negatively Regulates the ABA Response and Drought Tolerance by Down-Regulating AtMPK6-Related Pathway in *Arabidopsis*

**DOI:** 10.1371/journal.pone.0116385

**Published:** 2015-01-30

**Authors:** Dong-bei Xu, Ming Chen, Ya-nan Ma, Zhao-shi Xu, Lian-cheng Li, Yao-feng Chen, You-zhi Ma

**Affiliations:** 1 College of Agriculture, Northwest A&F University, Yangling, Shaanxi, 712100, China; 2 Institute of Crop Sciences, Chinese Academy of Agricultural Sciences (CAAS)/National Key Facility for Crop Gene Resources and Genetic Improvement, Key Laboratory of Biology and Genetic Improvement of *Triticeae* Crops, Ministry of Agriculture, Beijing, 100081, China; Texas Tech University, UNITED STATES

## Abstract

Heterotrimeric G-proteins are versatile regulators involved in diverse cellular processes in eukaryotes. In plants, the function of G-proteins is primarily associated with ABA signaling. However, the downstream effectors and the molecular mechanisms in the ABA pathway remain largely unknown. In this study, an *AGB1* mutant (*agb1-2*) was found to show enhanced drought tolerance, indicating that *AGB1* might negatively regulate drought tolerance in *Arabidopsis*. Data showed that AGB1 interacted with protein kinase AtMPK6 that was previously shown to phosphorylate *AtVIP1*, a transcription factor responding to ABA signaling. Our study found that transcript levels of three ABA responsive genes, *AtMPK6*, *AtVIP1* and *AtMYB44* (downstream gene of *AtVIP1*), were significantly up-regulated in *agb1-2* lines after ABA or drought treatments. Other ABA-responsive and drought-inducible genes, such as *RD29A* (downstream gene of *AtMYB44*), were also up-regulated in *agb1-2* lines. Furthermore, overexpression of *AtVIP1* resulted in hypersensitivity to ABA at seed germination and seedling stages, and significantly enhanced drought tolerance in transgenic plants. These results suggest that *AGB1* was involved in the ABA signaling pathway and drought tolerance in *Arabidopsis* through down-regulating the *AtMPK6*, *AtVIP1* and *AtMYB44* cascade.

## Introduction

Heterotrimeric GTP-binding proteins (G-proteins) are evolutionarily conserved plasma membrane-bound proteins that regulate a number of fundamental processes in eukaryotic organisms. G-proteins consist of three subunits, Gα, Gβ, and Gγ. In contrast to humans, which have 23 Gα, 5 Gβ and 14 Gγ genes [1], *Arabidopsis* has only one Gα (*GPA1*) [2], one Gβ (*AGB1*) [3] and three Gγ (*AGG1*-*AGG3*) genes [4,5]. Upon perception of a cognate ligand by its transmembrane G protein coupled receptor (GPCR) on the cell surface, the heterotrimeric G-protein complex dissociates to form an activated Gα-subunit and an obligate dimer Gβγ, which further transmits signaling into the cytoplasm by interaction with various downstream effector proteins [6]. Loss-of-function mutants and gain-of-function lines overexpressing G-protein subunits and signaling components showed that G-proteins play a vital role in regulating diverse growth and developmental processes, hormonal and stress responses [6,7]. AGB1 functions in many facets of development and signal transduction in *Arabidopsis*, for example, *agb1* mutants display diverse phenotypes with highly branched root systems, rounder leaves as well as shorter siliques [8], and have altered sensitivity to brassinosteroid (BR) and ABA during seed germination, and altered sugar sensing and stomate closure [9–13].

G proteins are involved in signal transduction through interaction with their effector proteins and regulate their activities [14]. Many G protein effectors have been functionally identified in animals, but few effectors for canonical G proteins were characterized, especially for AGB1 in plants. Currently, some genes involved in physical and genetic AGB1-interaction have been identified, such as ARD1 (ACI-reductone dioxygenase-like protein), a protein interacting with Gβ exhibited higher enzymatic activity by the involvement of Gβγ [15], NDL1 (N-MYC DOWNREGULATED-LIKE1), a protein physically interacting with AGB1 that regulates auxin distribution in roots [16], and a Golgi-localized hexose transporter, SGB1 [17], and an AGB1-interactome have been reported in *Arabidopsis* [18]. However, these studies have not explained the divergent functions of AGB1 in plants, and the molecular mechanisms underlying G protein-mediated ABA signaling remain to be investigated.

ABA is a crucial mediator in plant response to both biotic [19] and abiotic stresses, such as dehydration, salinity, low temperature [20,21], and in plant developmental processes such as seed development, dormancy, germination, and seedling growth [22]. Components of the heterotrimeric G-protein complex are involved in the ABA signaling pathway and play an important role in seed germination, early seedling development, stomate opening and closure in *Arabidopsis* [9,10,13,23,24]. In addition, ABA was shown to bind to GTG1 and GTG2 on the plasma membrane [25], and a quantitative proteomics-based analysis showed that many ABA-responsive proteins depend on the presence of functional GTG1/GTG2 proteins. This indicates the importance of G proteins in ABA signaling [26]. On the other hand, *agb1-2* mutant has greater ABA sensitivity than *gpa1-4* or *gcr1-2* mutants during seed germination and post-germination development, indicating that AGB1 is a primary regulator of the G-protein complex in ABA signaling [7,11]. However, the putative downstream effectors of AGB1 have not been assessed, therefore, the putative molecular mechanism underlying the involvement of *AGB1* in ABA signaling pathway remains unclear.

In this research, the role of *AGB1* in the ABA-related signaling pathway and its interaction proteins and/or downstream genes was investigated using yeast two-hybrids, and ABA-treated *Arabidopsis* cDNA library was screened using AGB1 as bait. AtMPK6 (mitogen-activated protein kinase 6) was found to interact with AGB1 [27]. Furthermore, the expression profiles of downstream genes in *agb1-2* mutant lines upon ABA and drought treatments were investigated. The results showed that a subset of genes involved in the ABA signaling pathway and in drought tolerance were up-regulated in *agb1-2* lines. Finally, the performance of transgenic *Arabidopsis* plants overexpressing *AtVIP1* (*VirE2-interacting protein1*), a gene encoding a bZIP transcription factor protein (a downstream target of *AtMPK6*), was investigated. Our results demonstrated that AGB1 interacts with AtMPK6 and may negatively regulate the ABA signaling pathway and drought tolerance by down-regulating the *AtMPK6, AtVIP1*, and *AtMYB44* cascade in *Arabidopsis*.

## Materials and Methods

### Plant materials and growth conditions

The mutants *agb1-1* and *agb1-2* in the Col-0 background have been described previously [8,9]. *agb1-2* (At4g34460) homozygous mutants were confirmed by genomic PCR using gene-specific primers P1: 5′ -TCATTAGATTGGACACCGGAG- 3′; P3: 5′ -TGTGAATCCTGCTGTAATCCC- 3′ and T-DNA border primer P2 (LBb1.3): 5′ -ATTTTGCCGATTTCGGAAC- 3′ (S1B Fig.). RT-PCR was performed using primers P1 and P3 (S1C Fig. and S2 Table). Homozygous mutant and wild-type seeds kept at identical conditions were surface sterilized with 30% bleach for 10 min and washed five times with sterile water. Seeds were plated on MS medium plates containing 3% sucrose in darkness for 3 d at 4°C, and then transferred to a growth chamber with a 16-h-light (20°C)/8-h-darkness (22°C) cycle.

### Yeast two-hybrid screening

For screening of proteins interacting with AGB1, the full coding region of *AGB1* was amplified using primers AtAGB1-BD-FULL-*Nde*I-F and AtAGB1-BD-FULL-*EcoR*I-R (S2 Table), and digested/ligated into the C-terminal of the Gal4 DNA binding domain in pGBKT7. The recombined vectors were co-transformed along with the ABA-treated *Arabidopsis* cDNA library into the yeast strain AH109 as described by the manufacturer (Clontech, USA; http://www.clontech.com/). To confirm interaction between AGB1 and AtMPK6, the full-length coding sequences of *AtMPK6* and *AGB1* were amplified with primers AtMPK6-BD-FULL-*Nde*I-F and AtMPK6-BD-FULL-*Sal*I-R, and AtAGB1-AD-FULL-*Nde*I-F and AtAGB1-AD-FULL-*EcoR*I-R (S2 Table). *AtMPK6* and *AGB1* were inserted into pGBKT7 and pGADT7 respectively. The two recombined vectors were co-transformed into the yeast strain AH109. Transformed yeast was cultivated in liquid SD/–Leu/–Trp to OD_600_ = 0.6, and then 5 μl of yeast suspension was added to each of the control SD/-Leu/-Trp and screening SD/–Leu/–Trp/–His/–Ade medium. The growth of yeast cells was photographed after 3 d of incubation at 30°C.

### Bimolecular fluorescence complementation (BiFC) assay

Full-length coding sequences of *AGB1* and *AtMPK6* were amplified from a cDNA library of *Arabidopsis* using gene-specific primers AtAGB1-YFP^N^-FULL-*Bam*HI-F and AtAGB1-YFP^N^-FULL-*Sal*I-R, and primers AtMPK6-YFP^C^-FULL-*Bam*HI-F and AtMPK6-YFP^C^-FULL-*Sal*I-R (S2 Table). *AGB1* sequence was inserted into the pSPYNE vector (fusing AGB1 to the N-terminal side of YFP) and the *AtMPK6* to the pSPYCE vector (fusing MPK6 to the C-terminal side of YFP) [28]. The empty vectors pSPYNE and pSPYCE, expressing split YFP domains were used as a negative controls, and the combination of bZIP63-YFP^N^ and bZIP63-YFP^C^ was used for a positive controls [28]. Both pSPYNE-AGB1 and pSPYCE-MPK6 were mixed, and co-transformed into protoplasts of *Arabidopsis* ecotype Col-0. Samples were incubated at 23°C for 16–20 h before observation. YFP fluorescence was recorded using a confocal laser-scanning microscope (Zeiss LSM700) and the nuclei of protoplasts were stained with 4’,6-diamidino-2-phenylindole (DAPI, Sigma).

### GST pull-down assay *in vitro*

The GST-AGB1 and His-MPK6 constructs were introduced into *E. coli* strain BL21 (DE3), inducing the expression of the fusion proteins by adding 1 mM of isopropylthio-β-galactoside (IPTG) at 37°C. The GST-AGB1 and His-MPK6 fusion proteins were purified using glutathione-agarose 4B (GE Healthcare) beads and Ni-agarose, respectively, according to the manufacturer’s instructions (GE Healthcare). For protein pull-down assays, the GST fusion proteins were bound to a glutathione sepharose 4B column and the loaded matrix was then incubated with purified His fusion protein in binding buffer [20 mM HEPES, pH 7.4, 1 mM EDTA, 5 mM MgCl_2_, 1 mM DTT, 0.1% Triton X-100, 1 mg ml^−1^ BSA and 1 mM PMSF] for 4 h at 4°C. The beads were then centrifuged at 2000 g for 1 min at 4°C and washed four times with protein pull-down wash buffer (1 × PBS: 137 mM NaCl, 8.1 mM Na_2_HPO_4_·12H_2_O, 2.68 mM KCl, 1.47 mM KH_2_PO_4_, 1 mM PMSF, pH 7.4). Bound proteins were eluted, and then fractionated by 10% SDS-PAGE, and analyzed by Western blotting using antibodies of HisProbe-HRP (Invitrogen).

### Co-immunoprecipitation (Co-IP) assay *in vivo*

*AGB1*, fused to Flag-tag (AtAGB1-Flag-FULL-F and AtAGB1-Flag-FULL-R, containing *Sal*I and *Spe*I restriction sites respectively), and the *AtMPK6*, fused to Myc-tag (AtMPK6-Myc-FULL-F and AtMPK6-Myc-FULL-R, containing *Sal*I and *Spe*I restriction sites respectively) were digested/ligated into pCAMBIA1300 vector (S2 Table). CsCl gradient centrifugation was used to purify the plasmids and the plasmids were transformed transiently into *Arabidopsis* mesophyII protoplasts (Clo-0). The protoplasts were homogenized overnight in buffer (10 mM Tris-HCl [pH 7.6], 150 mM NaCl, 2 mM EDTA, 0.5% [v/v] Nonidet P-40, 2 × protease inhibitor [Roche]) and then were centrifuged at 13,000 g for 15 min at 4°C. A 30 μl volume of anti-Myc agarose beads (Sigma-Aldrich) for immunoprecipitation of Myc-tag were incubated with the extraction supernatant for 3 h at 4°C. After the samples were washed five times with 1 ml of 1 × PBS buffer, the immunoprecipitated products were analyzed by Western blot. Antibodies of Anti-Myc (Sigma-Aldrich) or anti-Flag (Sigma-Aldrich) were used, and the chemiluminescence signal was detected by autoradiography.

### Subcellular localization analysis of proteins

Full-length cDNA fragments coding *AtMPK6* and *AGB1* were PCR-amplified with gene-specific primers AtMPK6-GFP-FULL-*Sal*I-F and AtMPK6-GFP-FULL-*Bam*HI-R, and AtAGB1-GFP-FULL-*Sal*I-F and AtAGB1-GFP-FULL-*Bam*HI-R (S2 Table), and inserted into the N-terminal side of 163h-GFP vector, respectively. Subcellular localization analysis of two fused proteins was completed in protoplasts of *Arabidopsis* ecotype Col-0 [29] and *agb1-2*. Localizations of MPK6-GFP and AGB1-GFP were recorded 15–18 h after transformation using a confocal laser-scanning microscope (Zeiss LSM700) and the nuclei of protoplasts were stained with 4’,6-diamidino-2-phenylindole (DAPI, Sigma).

### Generation of 35S::AtVIP1 transgenic *Arabidopsis*

The full-length coding sequence of *AtVIP1* was amplified with gene-specific primers AtVIP1-FULL-*Sma*I-F and AtVIP1-FULL-*Spe*I-R (S2 Table), and inserted into the binary vector pBI121 under control of the *Cauliflower Mosaic Virus* (CaMV) 35S promoter. The construct was confirmed by sequencing, and transformed into *Agrobacterium tumefaciens* strain (GV3101), and then transformed into wild-type *Arabidopsis* (Columbia-0 ecotype) by the floral dip method [30]. The seeds collected were then selected on MS medium containing 50 mgl^−1^kanamycin for three generations. Transgenic lines (T3 generation) with different expression levels of *AtVIP1* were used for further analysis.

### Stress tolerance analyses of *Arabidopsis* plants

Germination assay was performed using 60 seeds of the WT and OE-VIP1–4, 5, 6 lines (identical storage condition). Seeds were surface sterilized and plated on 0.8% agar (Sigma) containing MS salts (Sigma) and 3% (w/v) sucrose with or without ABA. Plated seeds were then chilled at 4°C in darkness for 3 d, and finally germinated at 22°C in 16 h light/8 h darkness. After 4 days, germination rates were scored based on emergence of the radical through the seed coat. This experiment was repeated three times.

For ABA sensitivity and PEG tolerance analyses, seeds of WT and OE-VIP1–4, 5, 6 lines were germinated on MS medium for 3 d, and then transferred to normal medium or medium contained ABA or PEG. Root lengths of seedlings grown in 16 h light/8 h darkness were recorded after 14 d. For drought tolerance analyses, three-week-old plants in soil were withheld from watering for 21 d and then were re-watered at the 21^st^ day of drought. Observations were recorded after 3 d or 5 d of re-watering.

### RNA extraction and gene expression analyses

21-d-old *Arabidopsis* plants (Col-0) and *agb1-2* mutant were removed from the soil and treated with 200 μM ABA (stock solution in ethanol) and intact plants were harvested 0.5, 1, 2, 4, 6, 8, 12, 16, and 24 h later. For dehydration stress, 21-d-old *Arabidopsis* plants and *agb1-2* mutant were removed from the soil, and placed on filter paper at room temperature, and intact plants were harvested 0.5, 1, 2, 4, 6, 8, 12, 16, and 24 h later. Total RNA was isolated and 2 μg of total RNA was used for first strand cDNA synthesis with a PrimeScript 1st Strand cDNA Synthesis kit (Takara). Quantitative expression assays were performed with the SYBR *Premix Ex Taq* (Takara) and an ABI 7300 according to the manufacturer’s protocols (Applied Biosystem). The PCR program was 95°C for 2 min followed by 40 cycles of denaturation for 15 s at 95°C and annealing/extension at 60°C for 1 min. Expressions of all genes were assayed for triplicated. Gene expression was calculated using the Delta-Delta cycle threshold method [31]. Relative quantitative results were calculated by normalization to *ACT2* (GenBank accession number: AT3G18780). The primer pairs used for real-time PCR are listed in S2 Table.

### Determination of proline content

The proline contents of wild type and *agb1-2* plants were determined as previously described [32,33].

## Results

### AGB1 negatively regulates ABA sensitivity and drought tolerance in *Arabidopsis*

**Figure 1:**
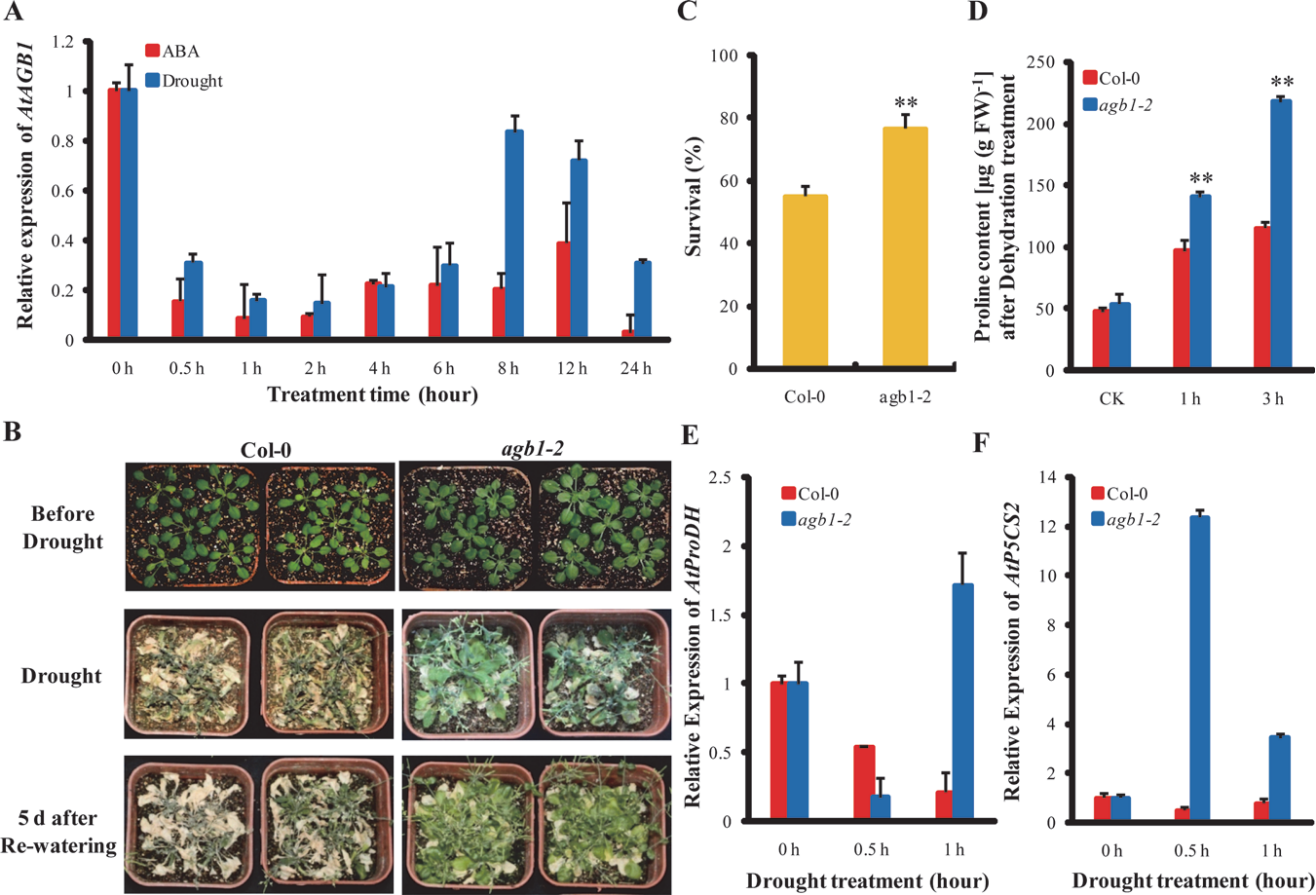
Expression pattern analysis of *AGB1* in *Arabidopsis* and drought tolerance assay of *agb1*. **(A)** Expression patterns of *AGB1* were identified after treatments with 200 μM ABA and drought stress for 0, 0.5, 1, 2, 4, 6, 8, 12, and 24 h. The expression value of *AGB1* at 0 h was normalized as 1; expression values at other time points were relative to the value at 0 h. Values represent means ± SD with three biological replicates. **(B)** Drought tolerance assay of WT (Col-0) and *agb1-2* plants. WT and *agb1-2* plants (before drought) were subjected to water stress by withholding water for 21 days (drought) and were then re-watered for 5 days (5 d after re-watering). **(C)** Survival rates of the WT and *agb1-2* in **(B)** after drought stress. Data represent means ± SD (n = 60) from three independent experiments. Asterisks indicate significant differences (Student’s t test, **P<0.01) between WT and *agb1-2*. **(D)** Proline contents of WT and *agb1-2* plants after dehydration treatments. For the proline assay, 14-day-old seedlings were subjected to drought treatment for 0, 1 and 3 h. Proline contents of WT and *agb1-2* plants at the same time points were significantly different at 1 and 3 h. Data represent means ± SD from three independent experiments. Asterisks indicate significant differences (Student’s t test, **P<0.01) between WT and *agb1-2*. **(E)** and **(F)** Expression analysis of the proline biosynthesis genes *ProDH* **(E)** and *P5CS2* **(F)** in WT and *agb1-2* seedlings after drought treatment for 0, 0.5 and 1 h. All results are means ± SD for three independent experiments.

Loss of function of *AGB1* increased ABA sensitivity either at seed germination and post-germination growth stages [11]. In the present study, the effects of ABA on the G-protein β subunit mutants *agb1-1* and *agb1-2* were examined. Under normal conditions without ABA treatment, the mutant *agb1-1, agb1-2* and WT lines showed 86–97% germination rates at 48 h (S2A Fig.) with no significant difference. Compared to the 72% germination rate of wild-type plants at 48 h, lower germination rates were observed in the *agb1-1* and *agb1-2* mutants, approximately 45% (for *agb1-1*) and 49% (for *agb1-2*), respectively, in the presence of 1.0 μM ABA (S2B Fig.). The cotyledon greening rates of WT, *agb1-1* and *agb1-2* seedlings were likewise similar under normal conditions, but those of WT were significantly higher than those in *agb1-1* and *agb1-2* seedlings growing on the medium supplemented with 1.0 μM ABA (*P<0.05) (S2 Fig. C and D), indicating that AGB1 plays a negative role in ABA signaling, similar to what was found previously [11,34]. Meantime, it was clear that the root lengths of *agb1* mutants were significantly shorter than that of wild-type under 2.0 μM ABA (*P<0.05) (S3 Fig. A, D and F), indicating that AGB1 may have a negative role in the ABA-mediated response. On the other hand, *AGB1* transcript was significantly decreased after treatment with 200 μM ABA (Fig. 1A) and also was drastically decreased after drought treatment with a slightly recovery at later stages (8 h and 12 h) (Fig. 1A), suggesting that *AGB1* was down-regulated under ABA and drought treatments.

To evaluate drought stress tolerance of G-protein β subunit mutant lines, we selected a knockout mutant *agb1-2*, and drought treated by withholding water from 3-week-old plants in pots for 3 weeks; 54.76% of WT plants survived for 5 d after re-watering, significantly lower than the 76.19% survival rate of *agb1-2* plants (**P<0.01) (Fig. 1B and C). Additionally, analysis showed that there were no differences in root length between wild-type (ecotype, Col-0) and *agb1* mutants on MS medium, however, in wild-type seedlings, roots length was significantly shorter than that of *agb1-1* and *agb1-2* mutant lines when the MS medium contained 4% and 8% PEG (*P<0.05) (S3 Fig. A, B, C and E). Physiological measurements showed that, under normal conditions, there was no significant difference in proline contents between WT and *agb1-2* (Fig. 1D). However, the proline contents in *agb1-2* plants were significantly higher at 1 h (31% higher) and 3 h (47% higher) than those in WT under dehydration at the same time point (**P<0.01) (Fig. 1D). Moreover, the expression levels of two key regulators of proline biosynthesis, *AtProDH* and *AtP5CS2*, were increased in *agb1-2* in comparison to WT plants after drought treatment (Fig. 1E and F), leading to proline accumulation and enhancement of drought resistance in *agb1-2* lines.

### AtMPK6 interacts with AGB1 in Y2H, pull-down, BiFC and CO-IP assays

**Figure 2:**
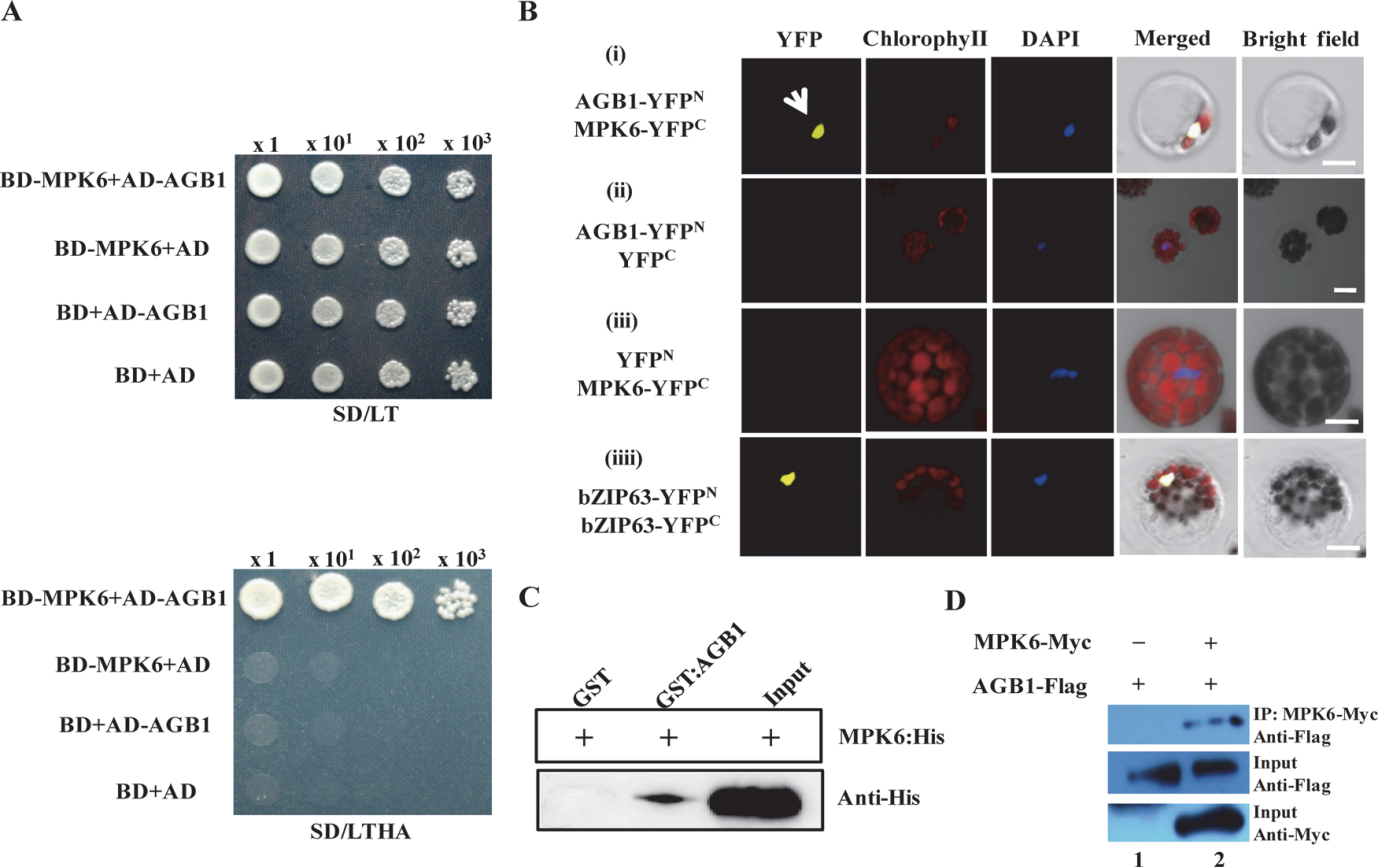
AtMPK6 interacted with AGB1 in Y2H, Pull-down, BiFC and Co-IP assays. **(A)** Interaction tests using yeast two-hybrid assays between AGB1 and AtMPK6. Yeast cells with AGB1 (AD-AGB1) and AtMPK6 (BD-MPK6) were placed in different liquid concentrations on control medium SD/-Trp/-Leu (SD/LT) and selection medium SD/-Trp/-Leu/-His/-Ade (SD/LTHA). For negative controls, pGBKT7 without insert (BD alone), pGADT7 without insert (AD alone), and the empty vectors AD and BD were used. Experiments were performed three times and a representative result is shown. **(B)** AGB1 interacted with AtMPK6 by BiFC assays in *Arabidopsis* protoplast. The recombinant constructs AGB1-YFP^N^ (YFP N-terminal) and MPK6-YFP^C^ (YFP C-terminal) were co-transformed into protoplast cells of *Arabidopsis* WT (Col-0). For negative controls, pSPYNE without insert AGB1 (YFP^N^) (iii) and pSPYCE without insert MPK6 (YFP^C^) (ii) were used. The combination of bZIP63-YFP^N^ and bZIP63-YFP^C^ was used as a positive control (iiii), Fluorescence was recorded 20 h after transformation. Experiments were performed 10 times and a representative result is shown. Bars = 40 μm in (i), (iii) and (iiii), and 20 μm in (ii). **(C)** Interaction assay using pull-down analysis. AGB1 and AtMPK6 fused with GST and His tags, respectively were mixed and passed through a glutathione column (binding GST tag). After elution with pull-down binding buffer, samples were separated by SDS-PAGE, and His-MPK6 was detected by immunoblotting with anti-His antibody. GST alone was used as the negative control. Experiments were performed three times and a representative result is shown. **(D)** Co-IP assay of AGB1 with AtMPK6. AGB1-Flag and MPK6-Myc or AGB1-Flag and empty pCAMBIA1300 vector (contained Myc-tags) were transiently co-transformed into *Arabidopsis* protoplast. After 16 h, the total Arabidopsis cell lysates were prepared for Co-IP with anti-Myc agarose. Then, anti-Myc immunoprecipitates were subjected to Western blot analysis with anti-Flag antibody (top). Meanwhile, the total cell lysates were also subjected to Western blot analysis with anti-Flag (middle), and anti-Myc (bottom, for MPK6-Myc expression) antibodies. Experiments were performed three times and a representative result is shown.

To identify proteins interacting with AGB1 in regulating the ABA signaling pathway, we performed a yeast two-hybrid (Y2H) screening of the ABA-treated *Arabidopsis* cDNA library by using the full-length AGB1 as bait. Yeast cells with both AtMPK6 (GenBank accession: AEC10325; AT2G43790) and AGB1 grew on the selection medium, but not when either was absent, indicating that AtMPK6 interacts with AGB1 in yeast cells (Fig. 2A). The interaction between these two proteins was further confirmed using a BiFC assay based on split YFP [28]. The N- and C-terminal domains of YFP were fused to AGB1 and AtMPK6, respectively, and transiently co-expressed in protoplasts of *Arabidopsis* ecotype Col-0. Fluorescence from reconstituted YFP indicated that interaction between AGB1 and AtMPK6 occurred in the nucleus (Fig. 2B). Interaction of these two proteins was also identified by GST pull down assays. Recombinant GST-AGB1 and His-AtMPK6 fusion proteins were purified and co-incubated with GST-binding beads. Western blot analysis further confirmed the interaction between AGB1 and AtMPK6 *in vitro* (Fig. 2C).

The interaction between AGB1 and AtMPK6 *in vivo* was also verified by the Co-IP assay in *Arabidopsis* protoplasts. Both *MPK6-Myc* and *AGB1-Flag* constructs were transiently co-expressed in *Arabidopsis* protoplasts. The anti-Myc agarose beads were used to immunoprecipitate Myc-tag from the *Arabidopsis* cell lysates when co-expressed with MPK6-Myc and AGB1-Flag. Antibodies for Flag-tag were used to detect the AGB1-Flag fusion protein in immunoprecipitation products. Western blot analysis indicated that AGB1-Flag fusion protein was detected in the immunoprecipitated samples, but not detected in the control samples co-expressing the empty vector pCAMBIA1300 (contained Myc-tag only) and the recombination vector AGB1-Flag (compare lanes 1 and 2, top panel, Fig. 2D). This suggest that AtMPK6 could interact with AGB1 in *Arabidopsis* cells.

### ABA treatment and mutation of AGB1 did not affect the subcellular localization of AtMPK6

**Figure 3:**
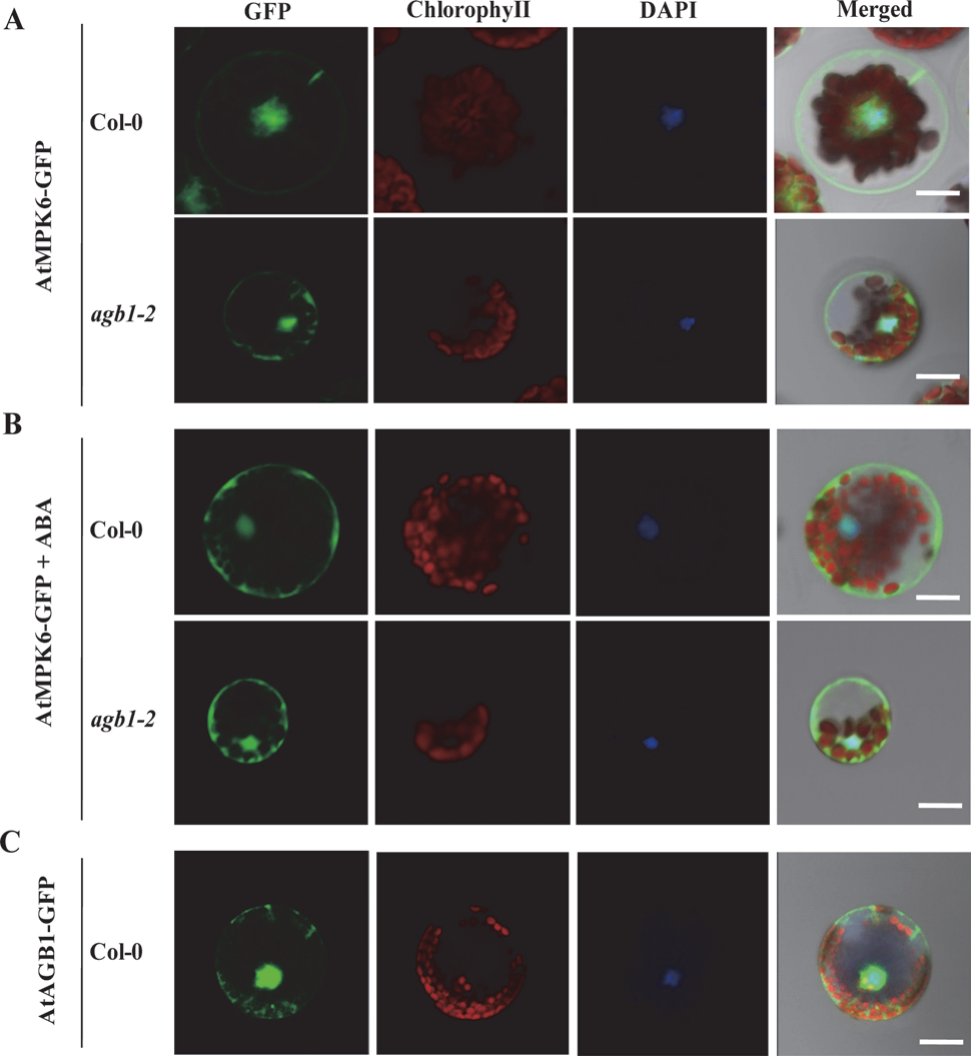
Subcellular localization analysis of AtMPK6 and AtAGB1. 35S:*AtMPK6*-GFP was transiently expressed in protoplast cells of *Arabidopsis* strains WT (Col-0) and the *agb1-2* mutant in **(A)** and **(B)**. Subcellular localizations of AtMPK6-GFP were detected without ABA treatment **(A)** and with 10 μM ABA **(B)**. Results were visualized by confocal microscopy. **(C)** Subcellular localization of AGB1. Each experiment was performed three times, and thirty cells were observed for each construct and a representative result is shown (Bars = 20 μm).

When transiently expressed in *Arabidopsis* protoplasts (ecotype Col-0), GFP-fused AtMPK6 (AtMPK6-GFP) was detected in the cytosol and nucleus. Similar results were obtained with *agb1-2* (Fig. 3A). When the protoplasts of both the mutant and wild type lines were treated with ABA, the localization of AtMPK6-GFP showed no difference (Fig. 3B), indicating that subcellular localization of AtMPK6 was unaffected by ABA treatment or mutation in *AGB1*. Additionally, our observation showed that AGB1 is present in the plasma membrane and nucleus (Fig. 3C), and it is identical with previous reports [34–36], suggesting that AtMPK6 and AGB1 co-localization in the nucleus might provide a novel insight into the interaction shown above by BiFC (Fig. 2B).

### AtVIP1, AtMYB44 and AtMPK6 were up-regulated in the *agb1-2* mutant after ABA or drought treatments

**Figure 4:**
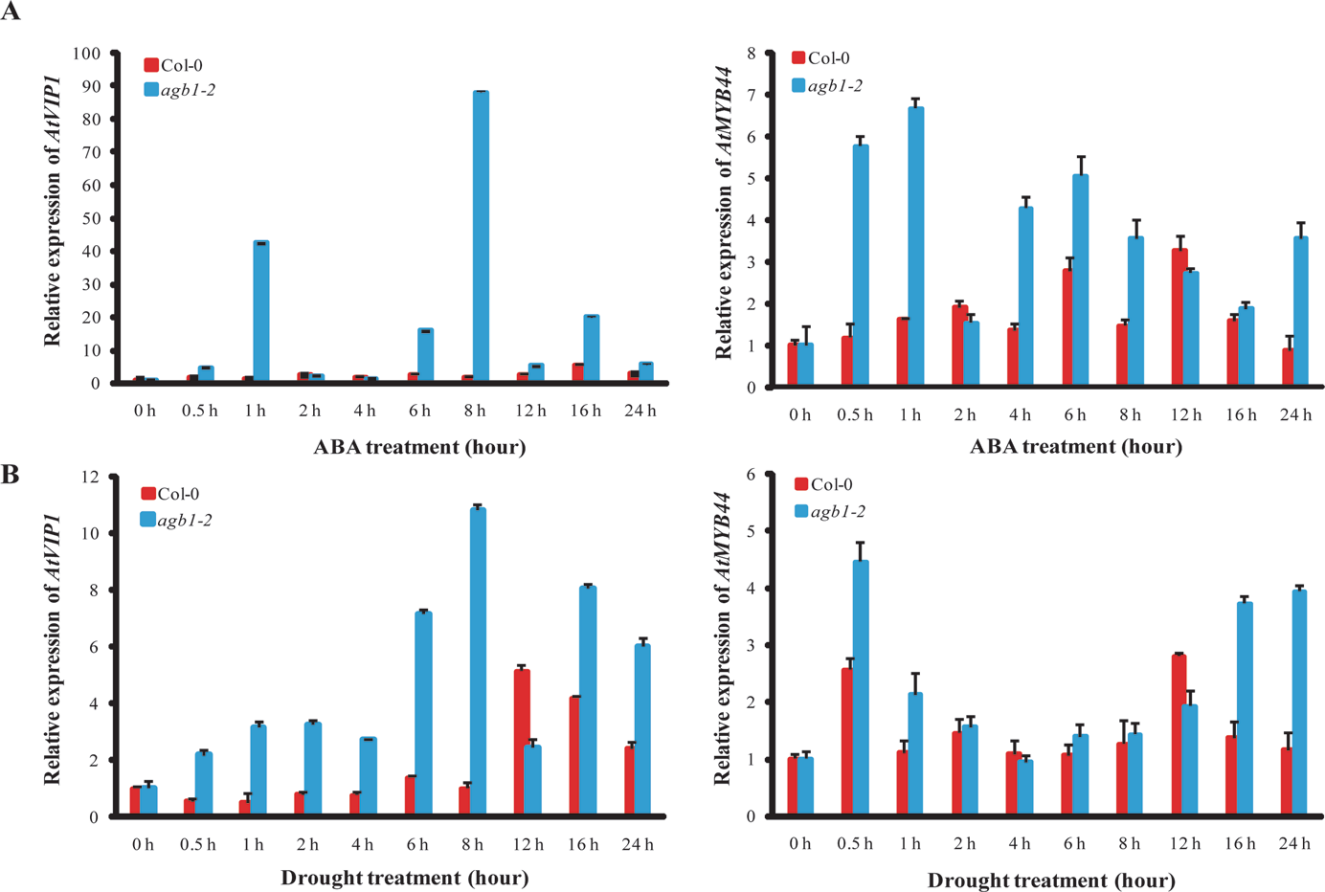
Expression of *AtVIP1* and *AtMYB44* in WT and *agb1-2* after ABA and drought treatments. **(A)** and **(B)** Expression analysis of *AtVIP1* and *AtMYB44* in WT and *agb1-2* plants after ABA **(A)** and drought **(B)** treatments for 0, 0.5, 1, 2, 4, 6, 8, 12, 16, and 24 h. Results are means ± SD with three biological replicates.

Both AtVIP1 and AtMYB44 can be phosphorylated by AtMPK6 [37,38]. Moreover, *AtVIP1* regulates expression of transcription factor *AtMYB44* [39] and overexpression of *AtMYB44* results in increased sensitivity of seed germination to ABA and enhances drought tolerance [40]. Therefore, expression of *AtVIP1* and *AtMYB44* were compared in *agb1-2* and WT plants subjected to ABA or drought treatments. Transcript levels of *AtVIP1* in *agb1-2* lines were significantly higher than those in WT, and reached nearly 35-fold and 51-fold at 1 h and 8 h, respectively, and that of *AtMYB44* reached nearly 5-fold and 4-fold at 0.5 h and 1 h in *agb1-2* lines under ABA treatment compared with WT (Fig. 4A). Except at 12 h, transcripts of *AtVIP1* in *agb1-2* lines were also higher than those in WT, and reached 10-fold at 8 h in *agb1-2* relative to WT, and transcripts of *AtMYB44* in *agb1-2* lines under drought treatment were 2.7-fold and 3.5-fold compared with that of WT at 16 h and 24 h, respectively (Fig. 4B). On the other hand, transcripts of *AtMPK6* in *agb1-2* lines were significantly increased at 0.5 h and 1 h after ABA treatment compared to those in WT (S4 Fig.). These results indicated that *AGB1* might negatively regulate the ABA signaling pathway in *Arabidopsis* by down-regulating expression of *AtMPK6, AtVIP1* and *AtMYB44* under ABA or drought treatments.

### A set of stress-responsive genes and ABA-biosynthesis genes were up-regulated in *agb1-2* after drought treatment

**Figure 5:**
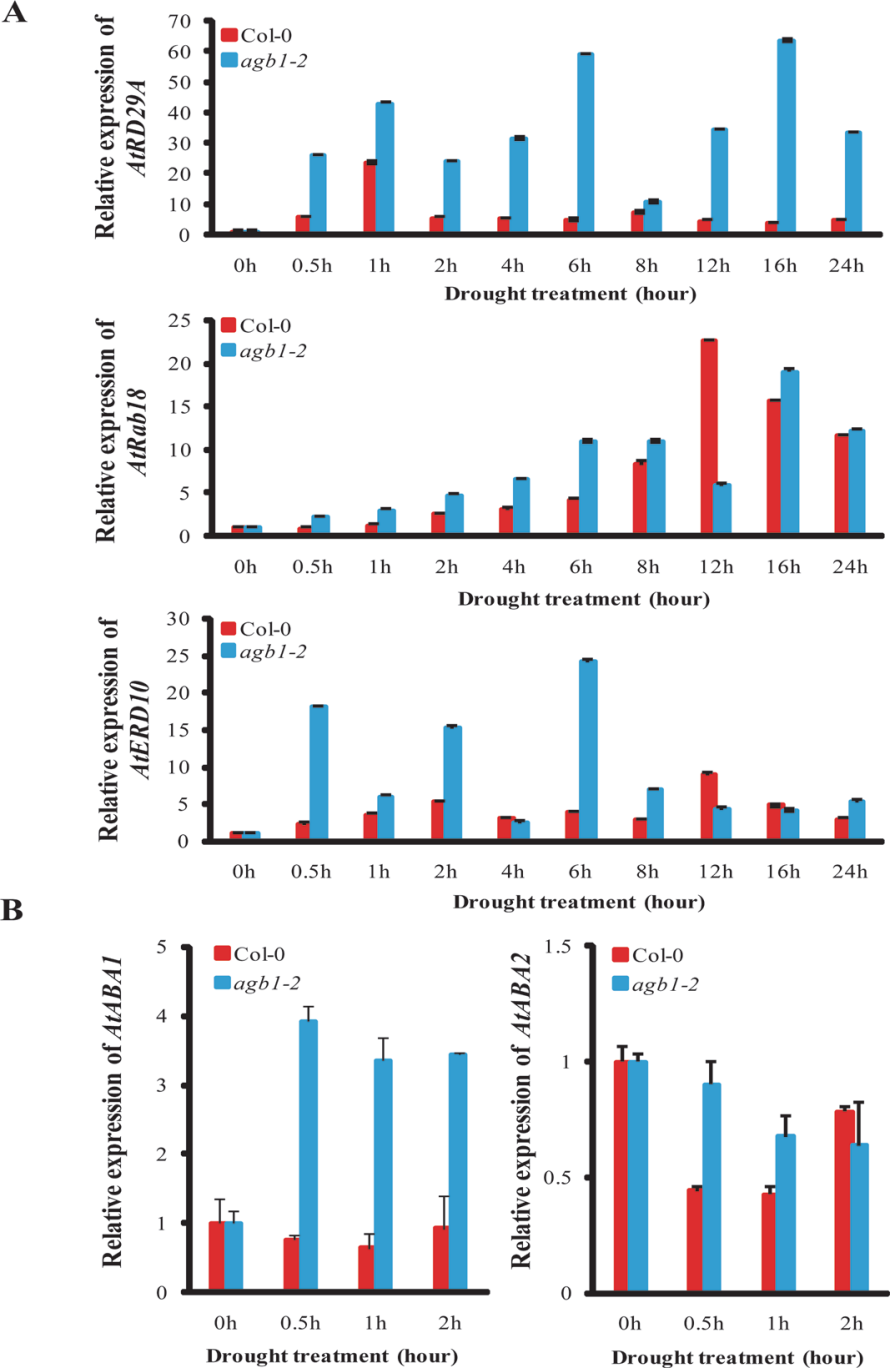
Expression of stress-responsive and ABA-biosynthesis genes in WT and *agb1-2* plants after drought treatment. **(A)** Expression of *RD29A, RAB18*, and *ERD10* in WT and *agb1-2* after drought treatment for 0, 0.5, 1, 2, 4, 6, 8, 12, 16, and 24 h. **(B)** Expression of ABA biosynthesis genes *ABA1* and *ABA2* in WT and *agb1-2* after drought treatment for 0, 0.5, 1, and 2 h. Results are means ± SD with three biological replicates.

Real-time PCR was performed to analyze the expression of *RD29A*, a downstream gene of *AtMYB44* [40], and stress-responsive genes *RAB18* and *ERD10* [41,42] in *agb1-2* and WT plants. When subjected to dehydration stress treatments, a set of stress-responsive genes, such as *RD29A, RAB18* and *ERD10* in WT and *agb1-2* were induced. For example, the expression of *RD29A* in *agb1-2* was higher than those in WT, reaching nearly 17-fold at 16 h after drought treatment compared with WT (Fig. 5A). Except at 12 h, the transcript levels of *RAB18* and *ERD10* in *agb1-2* were higher than those in WT, and the transcript levels of *RAB18* reached nearly 3-fold and that of *ERD10* nearly 6.5-fold higher at 6 h in *agb1-2* compared with WT (Fig. 5A). ABA-biosynthesis genes, such as *ABA1* and *ABA2*, were also up-regulated in *agb1-2* after drought treatment, and the expression of *ABA1* in *agb1-2* reached nearly 5.2-fold compared with that of WT at 1 h (Fig. 5B). These results indicated that *AGB1* negatively regulated the expression of a subset of drought-responsive genes involved in ABA signaling pathway, including *RD29A, RAB18, ERD10, ABA1* and *ABA2*.

### Transgenic 35S:*AtVIP1* plants were hypersensitive to ABA treatment and enhanced drought tolerance

**Figure 6:**
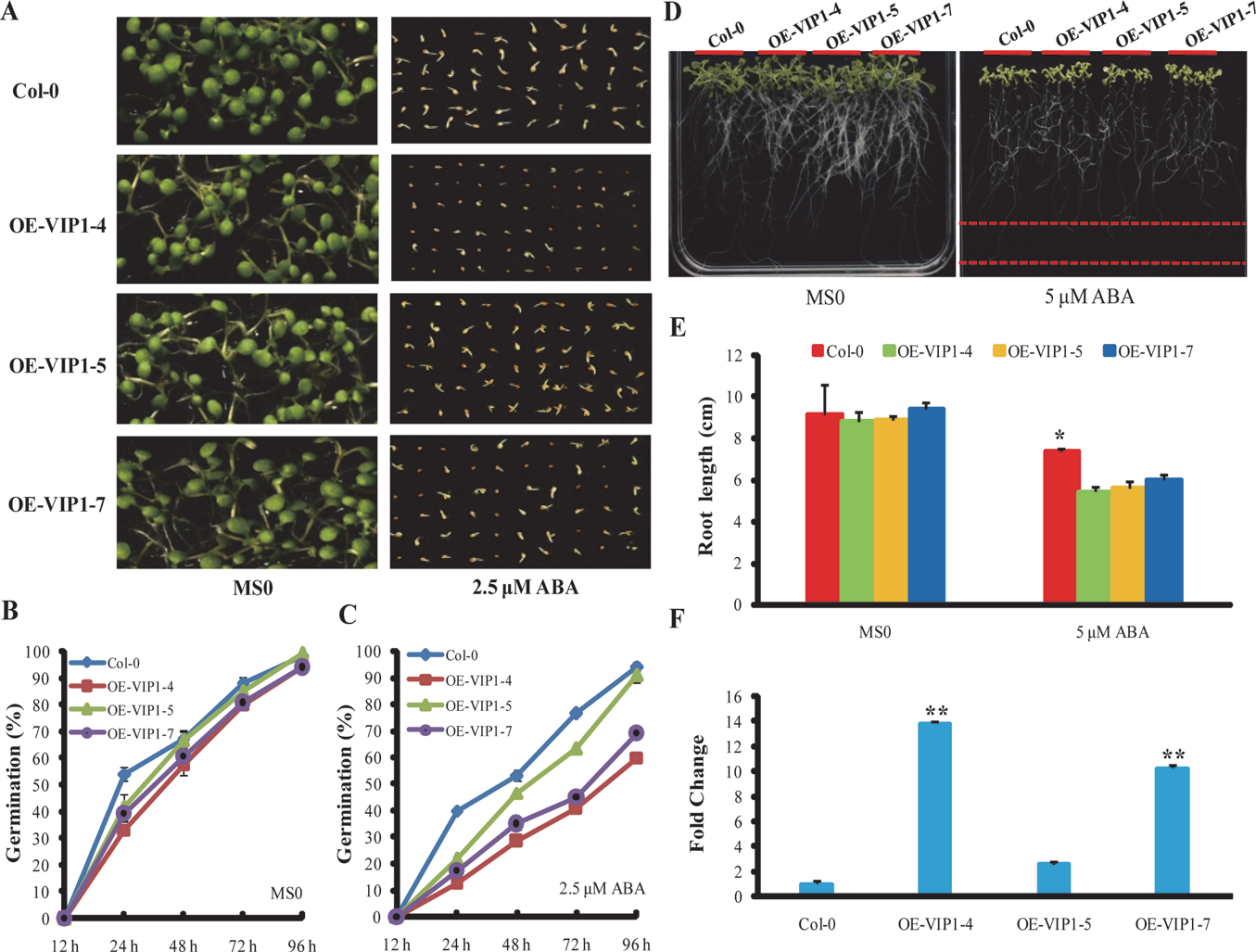
Overexpression of *AtVIP1* inhibited seed germination and root length under ABA treatment. Identically stored seeds were surface sterilized and washed extensively with water and plated on MS0 medium plates containing 3% Suc in the absence or presence of 2.5 μM ABA. Plates were kept at 4°C in darkness for 3 d and then transferred to growth chambers (16 h light/8 h darkness regime) at 22°C. **(A)** Seed germination analysis of WT and *35S:AtVIP1* transgenic plants containing OE-VIP1–4, -5 and -7 under normal conditions (MS0) and ABA treatment (MS0 plus 2.5 μM ABA). **(B)** and **(C)** Seed germination rates of different *35S:AtVIP1* transgenic lines under normal conditions **(B)**, and 2.5 μM ABA treatment **(C)** at different time points. *35S:AtVIP1* transgenic lines displayed lower germination rates than WT. Values for each time point are means of three experiments, and each experiment comprised 60 plants. **(D)** Root growth WT and *35S:AtVIP1* transgenic plants under normal conditions (MS0) and 5 μM ABA treatment for 14 d. **(E)** Primary root lengths (cm) shown in **(D)** were measured 14 days after treatment. Values are means ± SD (n = 30) from three independent experiments. Asterisk indicates significant differences (Student’s t test,*P<0.05) between the Col-0 and *35S:AtVIP1* transgenic lines. **(F)** Relative expression analysis (fold change) of *AtVIP1* in WT and transgenic lines. Expression of *VIP1* in WT was normalized as 1. Results are means ± SD from three independent experiments, and asterisks indicate significant differences (Student’s t test,**P<0.01) between Col-0 and transgenic lines.

**Figure 7:**
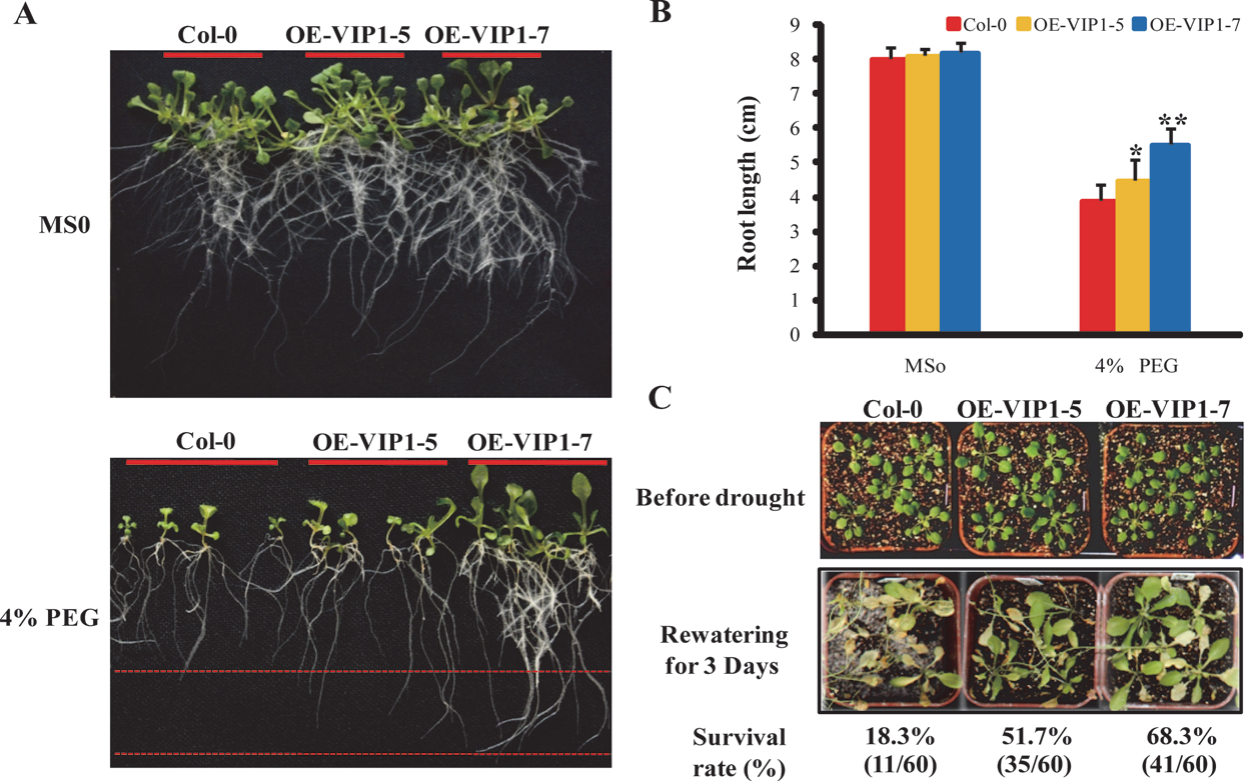
Drought tolerance analysis of *35S:AtVIP1* transgenic plants. **(A)** Growth of WT and *35S:AtVIP1* transgenic lines containing OE-VIP1–5 and -7 under normal conditions (MS0) and drought stress (MS0 plus 4% PEG). **(B)** Primary root lengths (cm) were measured at 14 days after PEG treatment. Values are means ± SD (n = 30) from three independent experiments and asterisks indicate significant differences (Student’s t test,*P<0.05 or **P<0.01) between WT and *35S::AtVIP1* transgenic plants. **(C)** Drought tolerance assay of the WT and *35S::AtVIP1* transgenic lines in soil. WT plants and *35S:AtVIP1* transgenic lines (21 days old, before drought treatment) were withheld from watering for 21 days and then re-watered for 3 days. Values under the figures are survival rates. Three independent experiments were conducted.

ABA functions to inhibit seed germination and seedling growth. Under normal conditions without ABA treatment, the *35S:AtVIP1* and WT lines showed similar germination rates (80–88%) at 72 h. However, the germination rates of *35S:AtVIP1* lines were 40–63% compared to 76% in WT at the same time point following treatment with 2.5 μM ABA (Fig. 6A, B and C). The root lengths of WT and *35S:AtVIP1* seedlings were likewise similar under normal conditions, but those of WT were significantly longer than *35S:AtVIP1* seedlings when grown on medium supplemented with 5 μM ABA (*P<0.05) (Fig. 6D and E). Germination rate of transgenic lines OE-VIP1–4 (40%) and OE-VIP1–7 (45%) were lower than that of line OE-VIP1–5 (63%) (Fig. 6C) after treatment with 2.5 μM ABA and the phenotypes of the transgenic lines were related to the expression levels of *AtVIP1* (Fig. 6F), indicating that the expression level of the *AtVIP1* transgene was positively related with sensitivity to ABA treatment in transgenic plants during germination and early seedling growth. With regard to osmotic stress, there were no differences between WT and *35S:AtVIP1* lines on MS medium, but the root lengths of WT seedlings were significantly shorter than those of *35S:AtVIP1* lines OE-VIP1–5 (P<0.05) and OE-VIP1–7 (P<0.01) when grown on MS medium contained 4% PEG (Fig. 7A and B). For plants grown in soil, *35S:AtVIP1* lines OE-VIP1–5 (51.7% survival) and OE-VIP1–7 (68.3% survival) retained better appearance and higher survival rates than WT (18.3% survival) at 3 d after re-watering following 21 d of water deprivation (Fig. 7C), suggesting that overexpression of *AtVIP1* conferred drought tolerance in transgenic plants.

## Discussion

### AGB1 negatively regulates the ABA response by down-regulating AtMPK6-AtVIP1-AtMYB44 pathway in *Arabidopsis*

The downstream effectors of G proteins and associated signaling pathways were not well-defined. AGB1 was previously considered to be a primary regulator of the G-protein complex in ABA signaling [7,11], whereas the regulatory mechanism of AGB1 in ABA responses in *Arabidopsis* remained unclear [11]. Recently, although several AGB1 interaction proteins in *Arabidopsis* have been identified in the G-signaling Interactome Database (AGIdb, http://bioinfolab.unl.edu/AGIdb), few protein kinases have been shown to interact with AGB1 [43,44]. In the present study, we used Y2H, Pull-down (*in vitro*), BiFC and Co-IP assay (*in vivo*) to demonstrate that AGB1 interacts with AtMPK6, a key protein kinase involved in stress responses (Fig. 2). These results established the link between G-proteins and the MAP-like kinase cascade. Protein kinases are major components in cellular signal transduction pathways, and mediate responses to various biotic and abiotic stresses, including hormone signaling pathways [45]. For example, *AtMPK6* was transiently activated after ABA application and overexpression of *AtMPK6* enhanced ABA response during germination. However, the *atmpk6* mutant was insensitive to ABA during germination and under drought stress condition [27,46,47], suggesting that the interaction between MPK6 and AGB1 may play a role in ABA signaling. In addition, both AtVIP1 and AtMYB44 can be phosphorylated by AtMPK6 [37,38]. *AtMYB44* is regulated by *AtVIP1* [39], and the dehydration-responsive gene, *RD29A*, is a downstream target gene of *AtMYB44* [40]. Furthermore, overexpression of *AtMYB44* also enhanced ABA sensitivity and drought tolerance in *Arabidopsis* [40]. The evidence suggested that a signaling cascade that involves *AtMPK6, AtVIP1, AtMYB44*, and *RD29A* might be involved in the ABA signaling pathway in *Arabidopsis*. In this report, the *agb1* mutants showed hypersensitivity to ABA (S2 Fig.), and enhanced drought tolerance (Fig. 1B and C). On the other hand, the target genes involved in this pathway were up-regulated in *agb1-2* under ABA or drought treatments (Fig. 4, Fig. 5A and S4 Fig.). Overexpression of *AtVIP1* enhanced the sensitivity of transgenic plants to ABA treatment (Fig. 6A-E). These results indicating that a novel role of AGB1 may be involved in transcriptional regulation through the *AtMPK6, AtVIP1, AtMYB44*, and *RD29A* cascade in *Arabidopsis* in response to ABA or dehydration.

With respect to the mechanism of AtMPK6 regulation by AGB1, it was reported that the activity of the kinase was regulated by affecting trafficking in plant cells [48]. However, the results showed that the subcellular localization of AtMPK6 was not affected in *agb1-2* (Fig. 3A), suggesting that trafficking changes were not involved in the regulation of AtMPK6 by AGB1. On the other hand, although the activity of AtMPK6 is reported to be independent of ABA [49], other studies have suggested that *AtMPK6* was transiently activated after ABA treatment in a AtMKK1-dependent manner in seedlings [27,46]. In the present study, the expression of *AtMPK6* in the *agb1-2* mutant was up-regulated after ABA application (S4 Fig.), implying that AGB1 might be involved in transcriptional regulation of *AtMPK6* in response to ABA. Whilst, other components might be involved in response to ABA signaling through interaction between AGB1 and AtMPK6. Recently, AtVIP1 was shown to interact with AGB1 in *Arabidopsis* [50] and modulated the osmo-sensing of *Arabidopsis* plants [51], and *AtVIP1* or *AtMYB44* acts as a downstream gene that can be regulated by *AtMPK6* [37,38]. Thus, AGB1 probably mediates the interaction of AtMPK6 and other components existing in the signaling complex in plant cells. This was due to WD40 repeats are thought to mediate protein-protein interactions, and AGB1 is one of these interaction proteins (http://smart.embl-heidelberg.de/). Additionally, some WD40 proteins in *Arabidopsis* are involved in protein degradation. Whilst, AGB1 interacts with an E3 ubiquitin ligase PUB20 and DDB1 [50,52,53]. So this does not rule out the possibility that the stability of AtMPK6 or its downstream substrates is regulated by AGB1. Previous reports showed that GPA1 interacted with PLDα1 and inhibited its activity by coupling with GDP [54]. ACI-reductone dioxygenase 1 (ARD1) interacted with AGB1, and was activated by AGB1 in *vitro* [15]. Although AGB1 did not affect the kinase activity of AtMPK6, and was not phosphorylated by AtMPK6 (data not shown), it will be interesting to examine whether AGB1 regulates the AtMPK6-dependent phosphorylation of MPK6 substrates.

In this study, we found that, except for *RD29A*, some stress-responsive genes such as *RAB18* and *ERD10* (Fig. 5A), ABA-biosynthesis genes containing *ABA1* and *ABA2* (Fig. 5B), and proline-biosynthesis genes including *AtProDH* and *AtP5CS2* (Fig. 1E and F) in *agb1-2* were significantly up-regulated after drought treatment, suggesting that in addition to the *AtMPK6, AtVIP1, AtMYB44*, and *RD29A* cascade, other factors might also be involved in ABA response and drought tolerance of plants regulated by AGB1. It was reported that Gβ interacts with MAPK-like protein, PsMPK3, and responds to ABA treatment in *Pisum sativum* [43]. As PsMPK3 is highly homologous with AtMPK3 and AtMPK6, two MAPKs that are key nodal and functionally redundant factors in the ABA signaling pathway [55], we hypothesized that AGB1 acts as a molecular switch in regulating the ABA signaling pathway in plants by interacting simultaneously with AtMPK3 and AtMPK6. However, further studies are necessary to confirm this hypothesis.

### AGB1 negatively regulates a drought tolerance in *Arabidopsis*

**Figure 8:**
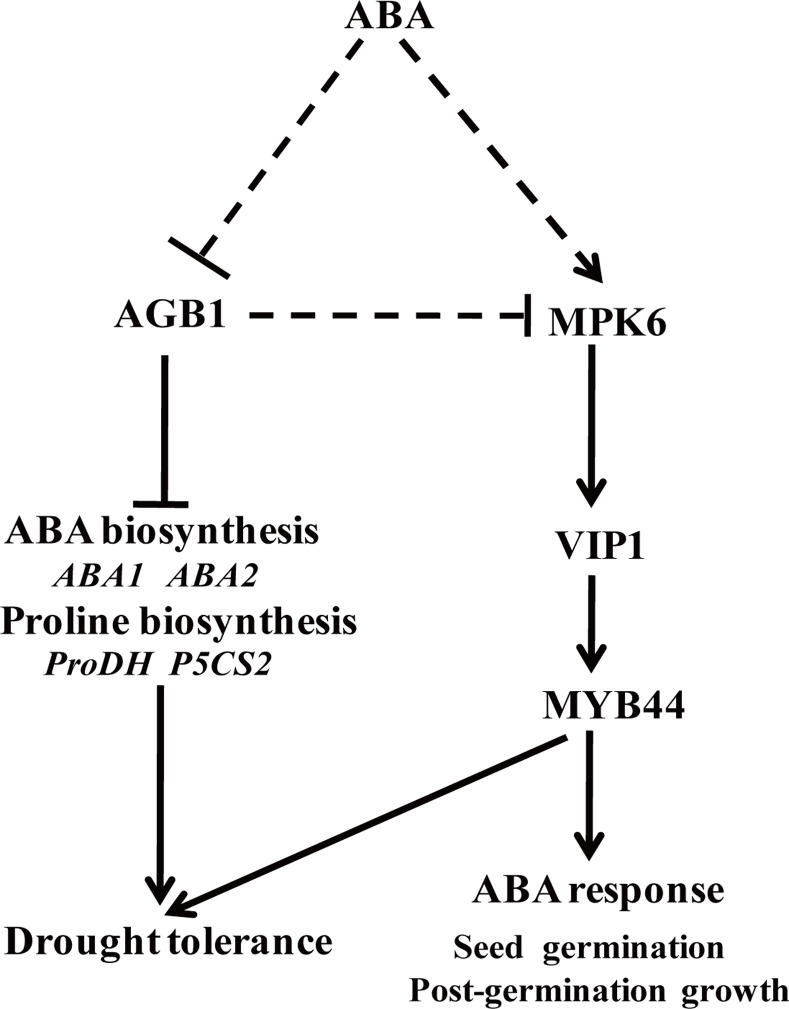
A mode for AGB1 functions in ABA- and drought stress response through affecting AtMPK6-related pathway. AGB1 negatively modulates ABA and drought response in *Arabidopsis* by down-regulating AtMPK6, AtVIP1 and AtMYB44 cascades and suppresses ABA biosynthesis and proline accumulation through regulating expression of *ABA1, ABA2, ProDH* and *P5CS2*. Positive effects are indicated by arrows and bars indicate repression.

*AGB1* not only affects the adaptability of plants to the environment, but also alters seed weight under water deficit [56]. However, the physiological and molecular mechanisms involved in the regulation of drought response are not completely understood. In this report, *AGB1* was demonstrated to negatively affect drought tolerance in *Arabidopsis* (Fig. 1B, C and S3E Fig.). Stress-responsive genes *RAB18* and *ERD10* (Fig. 5A), *ABA1* and *ABA2* (Fig. 5B), and *AtProDH* and *AtP5CS2* (Fig. 1E and 1F) in *agb1-2* were significantly up-regulated after drought, along with proline accumulation in the *agb1-2* mutant (Fig. 1D). It has been reported that overexpression of *AtMPK6* increased expression of the ABA biosynthesis genes *NCED3* and *ABA2* [47], and overexpression of stress-responsive genes, including *RD29A* and *RAB18*, which resulted in enhanced dehydration tolerance in transgenic plants [57,58]. Accumulation of proline is an important indicator in determining salt/drought tolerance, and overproduction of proline led to increased tolerance against abiotic stress in transgenic tobacco plants [59]. In this study, overexpression of *AtVIP1* enhanced drought tolerance of transgenic plants (Fig. 7A, B and C), indicating that inducible expression of stress-responsive genes contributed to enhanced drought tolerance in *agb1-2*. Moreover, we detected some cis-acting elements associated with abiotic stress responses, such as DRE (drought response element), LTRE (low temperature response element) and ABRE (ABA response element) in the promoter region of *AGB1* using the database PLACE Signal Scan Search (S1 Table) [60], suggesting that *AGB1* is indeed involved in drought responses in plants. Interestingly, *AGB1* expression was down-regulated by ABA and drought treatment (Fig. 1A). Therefore, it was hypothesized that *AGB1* negatively regulated expression of some ABA response and drought tolerance genes in wild type plants under normal conditions [40,61]. Whereas under stress conditions, expression of *AGB1* was inhibited (Fig. 1A), thereby expression of ABA response genes and drought tolerance genes were activated (Fig. 4A, B and Fig. 5A, B), thus contributing to enhanced stress tolerance and better survival under drought conditions. In brief, AGB1 interacts with AtMPK6 (Fig. 2A-D), and regulates ABA response and drought tolerance by down-regulating the ABA responsive transcriptional factor *AtMPK6* in *Arabidopsis* (Fig. 8).

## Conclusions

Suppression of *AtAGB1* enhanced ABA sensitivity and conferred drought tolerance in *Arabidopsis*, and AGB1 interacted with ABA-response protein kinase AtMPK6 *in vivo*. Moreover, the expression of three ABA responsive genes, *AtMPK6, AtVIP1* (downstream gene of *AtMPK6*) and *AtMYB44* (downstream gene of *AtVIP1*) were up-regulated in *agb1-2* mutant, and overexpression *AtVIP1* enhanced drought tolerance. All of these indicated that AGB1 regulated the AtMPK6-related pathway, but further studies are needed to determine the roles of AtMPK6 in AGB1-mediated intracellular signaling.

## Supporting Information

S1 Fig Identification of *AGB1* mutant. **(A)** Positions of T-DNA insertion in *agb1-2* mutant. Black boxes represent exons. The positions of T-DNA insertions in *agb1-2* is indicated by arrowheads. Primer pairs used in genomic PCR and primer pairs used in RT-PCR to assess *AGB1* transcripts are indicated. Annealing sites of the primers used in **(B)** and **(C)** are indicated by arrows. **(B)** Genomic PCR analyses verified homozygosity for the T-DNA alleles. **(C)** RT-PCR analysis of *AGB1* transcript levels in *agb1-2* mutant. *Actin* was used as an internal control.
(TIF)

S2 Fig *agb1* mutants showed increased sensitivity to ABA. Identically stored seeds were surface sterilized and washed extensively with water and plated on MS0 media plates containing 3% Suc in the absence or presence of 1.0 μM ABA. Plates were kept at 4°C in darkness for 3 d and then transferred to growth chambers (16 h light/8 h darkness regime) at 22°C. **(A)** and **(B)** Seed germination rates of wild-type (Col-0), *agb1-1* and *agb1-2* mutant lines under normal conditions **(A)**, and 1.0 μM ABA treatment **(B)** at different time points. Values for each time point are means of three experiments, and each experiment comprised 80 plants. **(C)** Photographs of greening seedlings of the wild type and *atagb1* grown on medium with or without 1.0 μM ABA after 21 d. **(D)** Cotyledon greening rates were calculated under normal condition and 1.0 μM ABA treatment for **(C)** experiment. Three experiments were performed with similar results. Values are means ± SD (n = 45). Asterisks indicate significant differences (Student’s t test,*P<0.05) between the Col-0 and *atagb1* mutant lines.
(TIF)

S3 Fig *agb1* mutants showed increased sensitivity to ABA and drought tolerance in seedling growth. Identically stored wild-type and *agb1* mutants seeds were surface sterilized and washed extensively with water and plated on MS0 medium plates containing 3% Suc. Plates were kept at 4°C in darkness for 3 d and then transferred to growth chambers (16 h light/8 h darkness regime) at 22°C. Seeds were germinated on MS0 medium for 3 d, and then transferred to normal medium **(A)**, MS0 medium plus 4% PEG **(B)** and 8% PEG **(C)**, and MS0 medium plus 2.0 μM ABA **(D)** for 11 d. **(E)** The length of primary roots was measured at 11 d after transfer corresponding to **(A, B, C)**. At least three experiments were done with similar results. Values presented are the mean ± SD (n = 20). Asterisks indicate a significant difference (Student’s t test, *p<0.05) between wild-type and *agb1* mutants. **(F)** The length of primary roots was measured at 11 d after transfer corresponding to **(D)**. Three experiments were done with similar results. Values presented are the mean ± SD (n = 20). Asterisks indicate a significant difference (Student’s t test, *p<0.05) between wild-type and *agb1* mutants.
(TIF)

S4 Fig The expression patterns of *AtMPK6*. Expression patterns of *AtMPK6* after 200 μM ABA treatment for 0, 0.5, and 1 h. The expression value of *AtMPK6* at 0 h was normalized as 1 for WT and *agb1-2*. Results are means ± standard deviation (SD, n = 3) and asterisks indicate significant differences (Student’s t test,*P<0.05 or **P<0.01) between WT and *agb1-2* at same time points.
(TIF)

S1 Table Analysis of cis-acting elements in *AGB1* promoter region. (DOC)

S2 Table Primers used in this study. (DOC)
