# Supplementary figures and images for: A G-Protein β Subunit, AGB1, Negatively Regulates the ABA Response and Drought Tolerance by Down-Regulating AtMPK6-Related Pathway in Arabidopsis
Source: PLoS One. 2015 Jan 30;10(1):e0116385. doi: 10.1371/journal.pone.0116385 (PMC4312036; doi:10.1371/journal.pone.0116385)

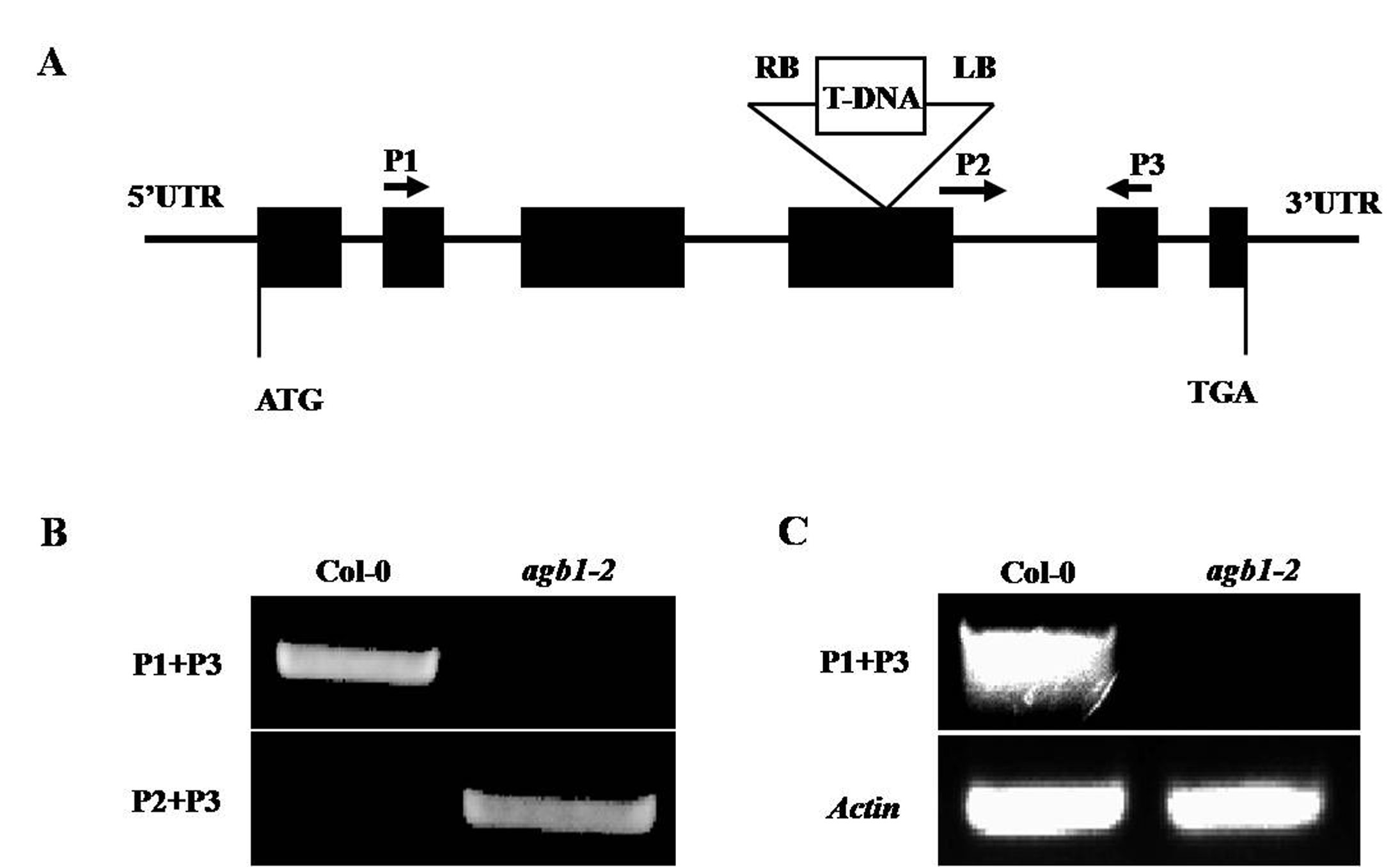

Supplement: S1 Fig — (A) Positions of T-DNA insertion in agb1-2 mutant. Black boxes represent exons. The positions of T-DNA insertions in agb1-2 is indicated by arrowheads. Primer pairs used in genomic PCR and primer pairs used in RT-PCR to assess AGB1 transcripts are indicated. Annealing sites of the primers used in (B) and (C) are indicated by arrows. (B) Genomic PCR analyses verified homozygosity for the T-DNA alleles. (C) RT-PCR analysis of AGB1 transcript levels in agb1-2 mutant. Actin was used as an internal control. (TIF) [file pone.0116385.s001.tif]

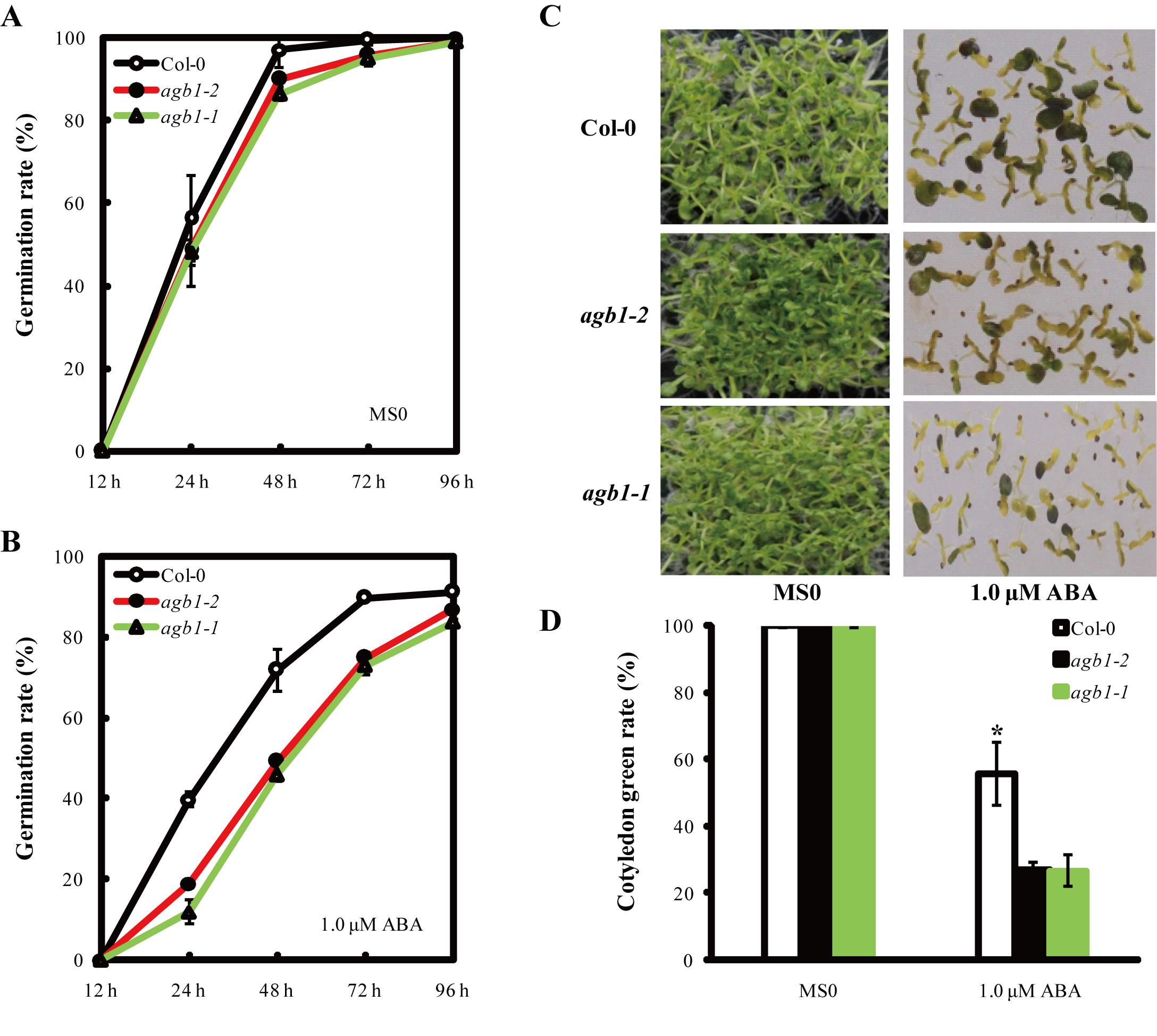

Supplement: S2 Fig — Identically stored seeds were surface sterilized and washed extensively with water and plated on MS0 media plates containing 3% Suc in the absence or presence of 1.0 μM ABA. Plates were kept at 4°C in darkness for 3 d and then transferred to growth chambers (16 h light/8 h darkness regime) at 22°C. (A) and (B) Seed germination rates of wild-type (Col-0), agb1-1 and agb1-2 mutant lines under normal conditions (A), and 1.0 μM ABA treatment (B) at different time points. Values for each time point are means of three experiments, and each experiment comprised 80 plants. (C) Photographs of greening seedlings of the wild type and atagb1 grown on medium with or without 1.0 μM ABA after 21 d. (D) Cotyledon greening rates were calculated under normal condition and 1.0 μM ABA treatment for (C) experiment. Three experiments were performed with similar results. Values are means ± SD (n = 45). Asterisks indicate significant differences (Student’s t test,*P<0.05) between the Col-0 and atagb1 mutant lines. (TIF) [file pone.0116385.s002.tif]

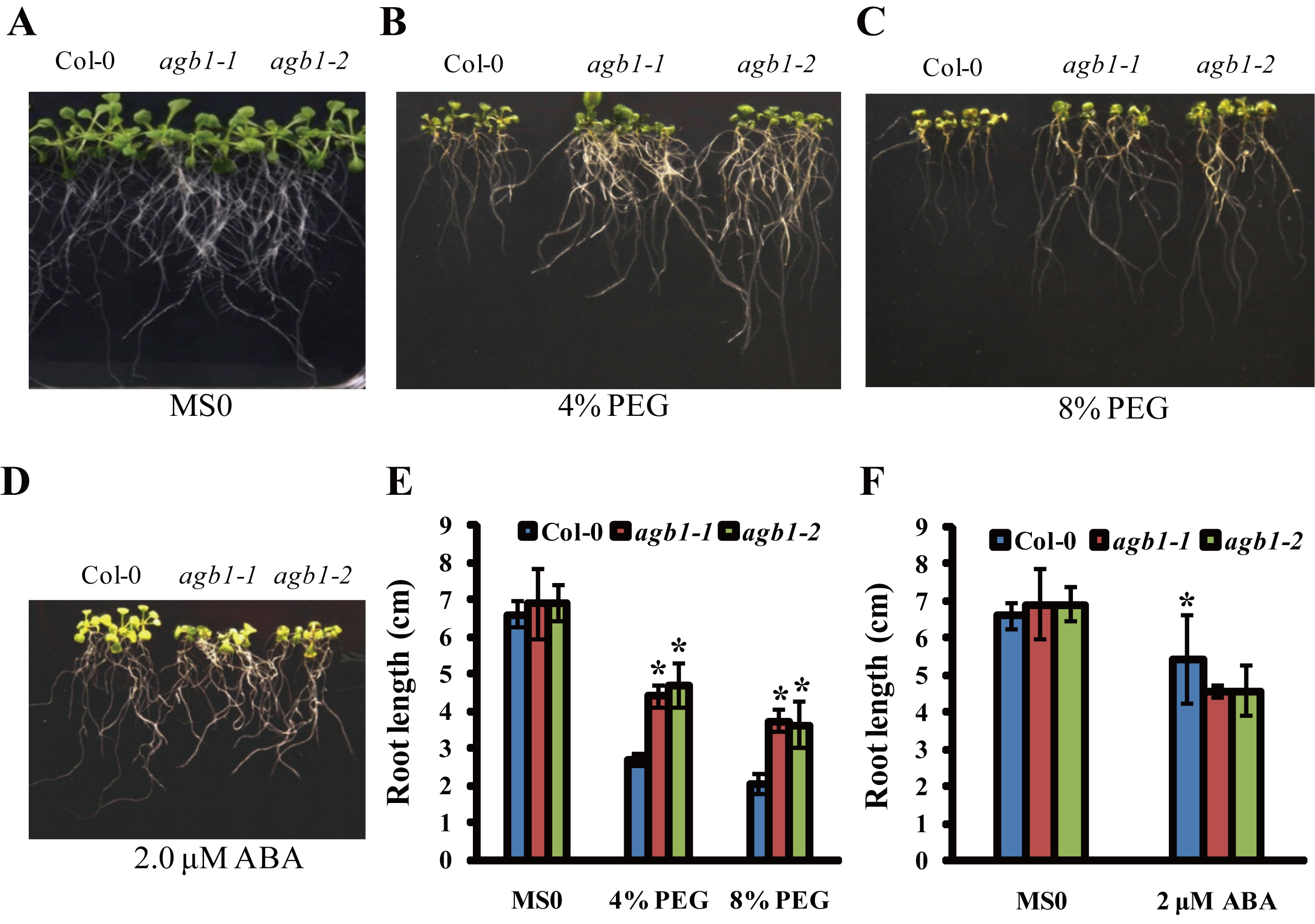

Supplement: S3 Fig — Identically stored wild-type and agb1 mutants seeds were surface sterilized and washed extensively with water and plated on MS0 medium plates containing 3% Suc. Plates were kept at 4°C in darkness for 3 d and then transferred to growth chambers (16 h light/8 h darkness regime) at 22°C. Seeds were germinated on MS0 medium for 3 d, and then transferred to normal medium (A), MS0 medium plus 4% PEG (B) and 8% PEG (C), and MS0 medium plus 2.0 μM ABA (D) for 11 d. (E) The length of primary roots was measured at 11 d after transfer corresponding to (A, B, C). At least three experiments were done with similar results. Values presented are the mean ± SD (n = 20). Asterisks indicate a significant difference (Student’s t test, *p<0.05) between wild-type and agb1 mutants. (F) The length of primary roots was measured at 11 d after transfer corresponding to (D). Three experiments were done with similar results. Values presented are the mean ± SD (n = 20). Asterisks indicate a significant difference (Student’s t test, *p<0.05) between wild-type and agb1 mutants. (TIF) [file pone.0116385.s003.tif]

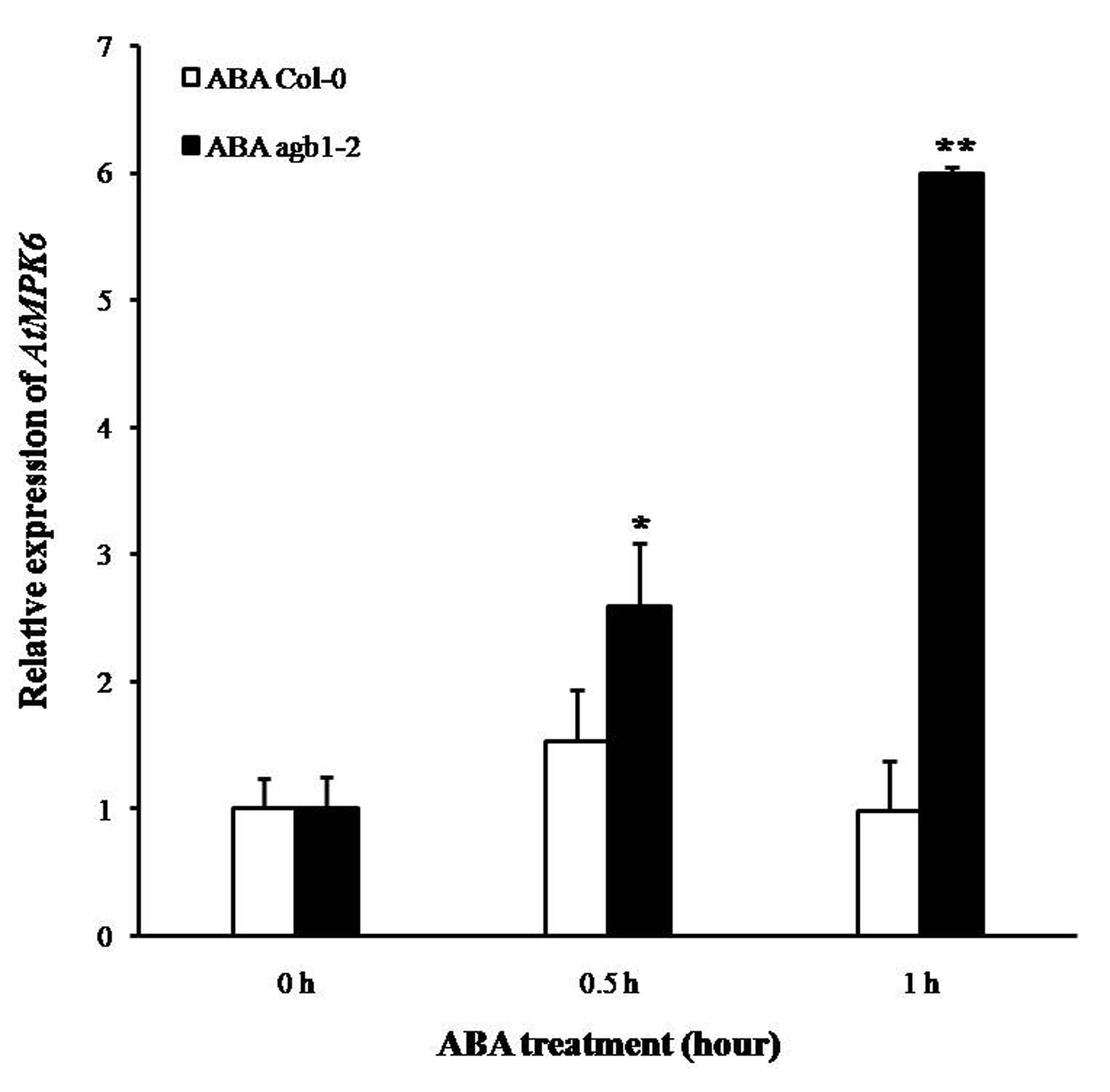

Supplement: S4 Fig — Expression patterns of AtMPK6 after 200 μM ABA treatment for 0, 0.5, and 1 h. The expression value of AtMPK6 at 0 h was normalized as 1 for WT and agb1-2. Results are means ± standard deviation (SD, n = 3) and asterisks indicate significant differences (Student’s t test,*P<0.05 or **P<0.01) between WT and agb1-2 at same time points. (TIF) [file pone.0116385.s004.tif]
