# Supplementary material for: A G-Protein β Subunit, AGB1, Negatively Regulates the ABA Response and Drought Tolerance by Down-Regulating AtMPK6-Related Pathway in Arabidopsis
Source: PLoS One. 2015 Jan 30;10(1):e0116385. doi: 10.1371/journal.pone.0116385 (PMC4312036; doi:10.1371/journal.pone.0116385)
Supplement: S1 Table — (DOC) [file pone.0116385.s005.doc]

**Table S1.** Analysis of cis-acting elements in *AGB1* promoter region. Cis-acting elements related to dehydration or ABA responses are in bold script

| Site name | Element sequence | No. of repeats | Function |
| --- | --- | --- | --- |
| NTBBF1ARROLB | ACTTTA | 2 | The DNA binding site of the Dof protein NtBBF1 is essential for tissue-specific and auxin-regulated expression of the rolB oncogene in plants |
| TAAAGSTKST1 | TAAAG | 2 | Found in promoter of KST1 (encodes a K1 influx channel in guard cells); target site for trans-acting StDof1 protein controlling guard cell-specific gene expression |
| CARGCW8GAT | CWWWWWWWWG | 8 | Binding site selection for the plant MADS domain protein AGL15 |
| WBOXATNPR1 | TTGAC | 4 | W-box found in the promoter of Arabidopsis NPR1 gene; recognized specifically by SA-induced WRKY DNA binding proteins |
| WBOXNTERF3 | TGACY | 6 | “W box” found in the promoter region of a transcriptional repressor ERF3 gene; involved in activation of ERF3 gene by wounding |
| REALPHALGLHCB21 | AACCAA | 2 | “REalpha” found in Lemna gibba Lhcb21 gene promoter; required for phytochrome regulation |
| MYB1AT | **WAACCA** | 3 | MYB recognition site found in the promoters of the dehydration-responsive gene rd22 and many other genes in Arabidopsis |
| MYCCONSENSUSAT | **CANNTG** | 9 | MYC recognition site found in the promoters of the dehydration-responsive gene rd22 |
| CBFHV | **RYCGAC** | 5 | Binding site of CBF1 in dehydration-responsive element (DRE) binding proteins (DREBs) |
| CRTDREHVCBF2 | GTCGAC | 4 | DNA-binding activity of an AP2 transcriptional activator HvCBF2 involved in regulation of low-temperature responsive genes in barley |
| MYBCORE | **CNGTTR** | 2 | Binding site for all animal MYB and at least two plant MYB proteins AtMYB1 and AtMYB2, both isolated from Arabidopsis; AtMYB2 is involved in regulation of genes that are responsive to water stress in Arabidopsis |
| DRE2COREZMRAB17 | **ACCGAC** | 1 | “DRE2” core found in maize (Z.M.) rab17 gene promoter; ABA responsiveness |
| DRECRTCOREAT | **RCCGAC** | 1 | Core motif of DRE/CRT (dehydration-responsive element/C-repeat) cis-acting element found in many genes in Arabidopsis and rice |
| LTRECOREATCOR15 | **CCGAC** | 1 | Core of low temperature responsive element (LTRE) of cor15a gene; ABA responsiveness |
| CIACADIANLELHC | CAANNNNATC | 5 | Region necessary for circadian expression of tomato (L.e.) Lhc gene |
| GT1GMSCAM4 | GAAAAA | 4 | "GT-1 motif" found in the promoter of soybean (*Glycine max*) CaM isoform, SCaM-4; Plays a role in pathogen- and salt-induced SCaM-4 gene expression |
| ABRELATERD1 | **ACGTG** | 3 | ABA response element-like sequence; required for etiolation-induced expression of erd1 (early responsive to dehydration) |
| CGACGOSAMY3 | CGACG | 2 | Found in the GC-rich regions of the rice Amy3D and Amy3E amylase genes; may function as a coupling element for the G box element |
| MYCATRD22 | **CACATG** | 1 | Binding site for MYC (rd22BP1) in Arabidopsis (A.t.) dehydration-responsive gene, rd22; ABA-induction |
| IBOXCORE | GATAA | 1 | I-box; conserved sequence upstream of light-regulated genes |
| ARFAT | TGTCTC | 1 | ARF (auxin response factor) binding site found in the promoters of primary/early auxin response genes of *Arabidopsis thaliana* (A.t.); AuxRE |
