# Supplementary material for: A G-Protein β Subunit, AGB1, Negatively Regulates the ABA Response and Drought Tolerance by Down-Regulating AtMPK6-Related Pathway in Arabidopsis
Source: PLoS One. 2015 Jan 30;10(1):e0116385. doi: 10.1371/journal.pone.0116385 (PMC4312036; doi:10.1371/journal.pone.0116385)
Supplement: S2 Table — (DOC) [file pone.0116385.s006.doc]

**Table S2. Primers used in this study**

| **Name Primer sequence (5’-3’)** | |
| --- | --- |
| **For *AGB1* mutant detected** | |
| P1 TCATTAGATTGGACACCGGAG | |
| P2 (LBb1.3) ATTTTGCCGATTTCGGAAC | |
| P3 TGTGAATCCTGCTGTAATCCC | |
| **For qRT-PCR analyses** | |
| qRT-AtAGB1-F | CTGGCTATGCGAGCAACAAC |
| qRT-AtAGB1-R | AGGCACTTCCATCTGCTGAC |
| qRT-AtProDH-F | TTCAAGTTGGTGAGAGGGGC |
| qRT-AtProDH-R | TTTCCTCGACGCAAGTCTCC |
| qRT-AtP5CS2-F | TACAGTTCCAAGGCCTGCAC |
| qRT-At P5CS2-R | CAGCACCAAGTCCGAACCTA |
| qRT-AtERD10-F | TCTCTGAACCAGAGTCGTTT |
| qRT-AtERD10-R | CTTCTTCTCACCGTCTTCAC |
| qRT-AtRAB18-F | CAGCAGCAGTATGACGAGTA |
| qRT-AtRAB18-R | CAGTTCCAAAGCCTTCAGTC |
| qRT-AtRd29A-F | ATCACTTGGCTCCACTGTTGTTC |
| qRT-AtRd29A-R | ACAAAACACACATAAACATCCAAAGT |
| qRT-AtMPK6-F | TTTGGGCTAGCTCGAGTCAC |
| qRT-AtMPK6-R | CCAGGGAAGAGTGGCTTACG |
| qRT-AtVIP1-F | ATGGAGAGATGAGTTCGGCG |
| qRT-AtVIP1-R | ATGGAGAGATGAGTTCGGCG |
| qRT-AtMYB44-F | TCCACCTGTTGTTACTGGGC |
| qRT-AtMYB44-R | TCCACCTGTTGTTACTGGGC |
| qRT-ACT2-F | GAAATCACAGCACTTGCACC |
| qRT-ACT2-R | AAGCCTTTGATCTTGAGAGC |
| **To construct vectors** | |
| AtAGB1-BD/AD-FULL-*Nde*I-F | GGAATTCCATATGATGTCTGTCTCCGAGCTCAAAG |
| AtAGB1-BD/AD-FULL-*EcoR*I-R | CGGAATTCTCAAATCACTCTCCTGTGTCCTC |
| AtMPK6-BD-FULL-*Nde*I-F | GGAATTCCATATGATGGACGGTGGTTCAGGTCA |
| AtMPK6-BD-FULL-*Sal*I-R | GCGTCGACGCTATTGCTGATATTCTGGATTG |
| AtAGB1-YFP^N^-FULL-*Bam*HI-F | CGGGATCCATGTCTGTCTCCGAGCTCAAAG |
| AtAGB1-YFP^N^-FULL-*Sal*I-R | GCGTCGACAATCACTCTCCTGTGTCCTC |
| AtMPK6-YFP^C^-FULL-*Bam*HI-F | CGGGATCCATGGACGGTGGTTCAGGTCA |
| AtMPK6-YFP^C^-FULL-*Sal*I-R | GCGTCGACTTGCTGATATTCTGGATTGA |
| AtAGB1-GST-FULL-F | CGGGATCCATGTCTGTCTCCGAGCTCAAAG |
| AtAGB1-GST-FULL-R | CCCTCGAGTCAAATCACTCTCCTGTGTCCTC |
| AtMPK6-His-FULL-F | CGGGATCCATGGACGGTGGTTCAGGTCA |
| AtMPK6-His-FULL-R | GCGTCGACGCTATTGCTGATATTCTGGATTG |
| AtMPK6-GFP-FULL-*Sal*I-F | GCGTCGACATGGACGGTGGTTCAGGTCA |
| AtMPK6-GFP-FULL-*Bam*HI-R | CGGGATCCTTGCTGATATTCTGGATTGA |
| AtAGB1-GFP-FULL-*Sal*I-F | GCGTCGACATGTCTGTCTCCGAGCTCAAAG |
| AtAGB1-GFP-FULL-*Bam*HI-R | CGGGATCCAATCACTCTCCTGTGTCCTC |
| AtAGB1-Flag-FULL-F | GGGGCCCGGGGTCGACATGTCTGTCTCCGAGCTCAAAG |
| AtAGB1-Flag-FULL-R | TACCGGATCCACTAGTAATCACTCTCCTGTGTCCTC |
| AtMPK6-Myc-FULL-F | GGGGCCCGGGGTCGACATGGACGGTGGTTCAGGTCA |
| AtMPK6-Myc-FULL-R | TACCGGATCCACTAGTTTGCTGATATTCTGGATTGA |
| AtVIP1-FULL-*Sma*I-F | CCCCCGGGATGGAAGGAGGAGGAAGAGGACCAAAT |
| AtVIP1-FULL-*Spe*I-R | GGACTAGTTCAGCCTCTCTTGGTGAAATCCATGTA |
